# Postoperative outcomes of resectable periampullary cancer accompanied by obstructive jaundice with and without preoperative endoscopic biliary drainage

**DOI:** 10.3389/fonc.2022.1040508

**Published:** 2022-11-10

**Authors:** Tanawat Pattarapuntakul, Tummarong Charoenrit, Nisa Netinatsunton, Thanapon Yaowmaneerat, Thakerng Pitakteerabundit, Bancha Ovartlarnporn, Siriboon Attasaranya, Thanawin Wong, Naichaya Chamroonkul, Pimsiri Sripongpun

**Affiliations:** ^1^ Gastroenterology and Hepatology Unit, Division of Internal Medicine, Faculty of Medicine, Prince of Songkla University, Hat Yai, Songkhla, Thailand; ^2^ Nanthana-Kriangkrai Chotiwattanaphan (NKC) institute of Gastroenterology and Hepatology, Faculty of Medicine, Prince of Songkla University, Hat Yai, Songkhla, Thailand; ^3^ HepatoPancreatoBiliary surgery unit, Department of Surgery, Faculty of Medicine, Prince of Songkla University, Hat Yai, Songkhla, Thailand

**Keywords:** preoperative biliary drainage, direct surgery, resectable periampullary cancer, obstructive jaundice, postoperative outcomes

## Abstract

**Background:**

Preoperative biliary drainage (PBD) is useful in resectable periampullary cancer with obstructive jaundice. Whether it is better than direct surgery (DS) in terms of postoperative complications and mortality is controversial.

**Methods:**

All cases of successful pancreaticoduodenectomy (PD) in patients with periampullary cancer with obstructive jaundice performed between January 2016 and January 2021 were retrospectively reviewed. Endoscopic PBD was performed; data pertaining to serum bilirubin level, procedural technique, and duration before surgery were obtained. The incidence of postoperative complications and survival rate were compared between the PBD and DS group.

**Results:**

A total of 104 patients (PBD, n = 58; DS, n = 46) underwent curative PD. The mean age was 63.8 ± 10 years and 53 (51%) were male. Age, body mass index (BMI), sex, Eastern Cooperative Oncology Group status, presence of comorbid disease, initial laboratory results, and pathological diagnoses were not significantly different between the two groups. The incidence of postoperative complications was 58.6% in the PBD group while 73.9% in the DS group (relative risk [RR] 1.26, 95% confidence interval [CI] 0.92, 1.73, p=0.155) and the difference was not significant except in bile leakage (RR 8.83, 95% CI 1.26, 61.79, p = 0.021) and intraoperative bleeding (RR 3.97, 95% CI 0.88, 17.85, p = 0.049) which were higher in the DS group. The one-year survival rate was slightly less in the DS group but the difference was not statistically significant. The independent predictors for death within 1-year were intraoperative bleeding and preoperative total bilirubin > 14.6 mg/dL.

**Conclusions:**

PBD in resectable malignant distal biliary obstruction showed no benefit in terms of 1-year survival over DS approach. But it demonstrated the benefit of lower risks of intraoperative bleeding, and bile leakage. Additionally, the level of pre-operative bilirubin level of over 14.6 mg/dL and having intraoperative bleeding were associated with a lower 1-year survival in such patients. Overall, PBD may be not necessary for all resectable periampullary cancer patients, but there might be a role in those with severely jaundice (>14.6 mg/dL), as it helps lower risk of intraoperative bleeding, and might lead to a better survival outcome.

## Background

Obstructive jaundice is the one of common presentations of periampullary cancers, and surgical resection is the curative treatment but could be applied only in the early-stage patients (1). Presence of obstructive jaundice is also a significant risk factor for postoperative complications attributed to impaired immune response, coagulopathy, kidney dysfunction, and impaired healing of anastomosis secondary to surgical site infection (2, 3). Therefore, preoperative biliary drainage (PBD) is potentially beneficial in lowering serum bilirubin level and reducing the incidence of subsequent complications, thereby preventing hepatobiliary dysfunction and improving the quality of life of patients (4). Previous meta-analyses showed comparable postoperative outcomes such as infection and mortality between those who underwent PBD and direct surgery (DS) (5, 6). However, conflicting outcomes of PBD including increasing risk of preoperative bacterial contamination of bile, cholangitis, and postoperative complications have also been reported (7–9). While the benefit of endoscopic biliary drainage in unresectable periampullary cancers with malignant obstruction is evidently concrete (10, 11), there are no current recommendations regarding the decision between PBD and DS for potentially resectable periampullary cancer patients.

Several factors might affect the outcomes of PBD. Theoretically, patients with a higher degree of jaundice may be benefit from PBD. A prior randomized controlled trial published in 2010 showed that PBD showed no benefit over early surgery in those with serum bilirubin level <14.6 mg/dL (1). nonetheless, whether PBD in patients with deeper jaundice will be beneficial over direct surgery is still unknown (1, 12). Moreover, the waiting duration for surgery, from PBD to surgery, has varied among studies, and has been reported as 2 weeks, 2-4 weeks, and > 4 weeks. A longer waiting duration seemed to increase the incidence of biliary complications and poor operative outcomes, especially infectious complications, over time (1).

The advantages of PBD in patients with periampullary cancer who present with obstructive jaundice at the resectable stage remain unclear. Most studies have been conducted in the United State of America and Europe, which have good healthcare referring systems, high socioeconomic statuses, and short waiting duration for surgery.

In this study, we aimed to evaluate postoperative outcomes in terms of postoperative complications, length of hospital stays, and mortality in patients with periampullary cancers who underwent curative surgical resection with or without PBD.

## Methods

We conducted a retrospective single center cohort study, which included patients with periampullary cancer who had undergone curative surgical resection (Whipple’s operation or pyloric preserving pancreaticoduodenectomy [PPPD]) with or without endoscopic PBD at our center between January 2016 and January 2021. Specifically, all endoscopic procedures were performed at Songklanagarind hospital, the largest university hospital in Southern Thailand. The inclusion criteria were as follows: 1) patients with periampullary tumor diagnosed by computed tomography (CT), magnetic resonance imaging (MRI) and considered to be resectable after an evaluation by hepato-pancreato-biliary (HPB) surgeons, 2) age of at least 18 years, 3) total bilirubin level at the time of diagnosis > 3 mg/dL, and 4) with complete follow-up data. Patients with disease progression to the unresectable stage or locally advanced stage during the waiting duration for surgery were excluded. Eligible patients were identified from our endoscopic center and HPB registration center’s database. All patients’ profiles and procedural data were extracted and collected from the hospital’s electronic database.

The study protocol was approved by the Institutional Review Board of Faculty of Medicine, Prince of Songkla University (REC 64-473-21-1). The need for informed consent from the participants was waived owing to the retrospective nature of the study.

### Resectability determination of periampullary cancer

Periampullary cancer was identified through CT, MRI and reviewed by experienced body radiologists. The tumor considered to be resectable if none of the following criteria were met: infiltration of peripancreatic fat planes, the hepatoduodenal ligament and the mesentery; > 180 ° encasement of the portal or superior mesenteric vein or of the hepatic or superior mesenteric artery; or distant metastasis (13).

At our institute, the decision to perform direct surgery (DS) or PBD in patients with resectable periampullary cancers presenting with obstructive jaundice is made by the attending physician. Generally, PBD is performed in patients with obstructive jaundice, ascending cholangitis, or prolonged waiting duration for surgery (2), but there were no pre-defined criteria for PBD in our institute, to drain or not drain prior to the surgery is as per the attending physician’s decision. According to the current practice in hepato-pacreato-biliary(HPB) surgery, definite tissue diagnosis is not mandatory before curative resection.

### Endoscopic biliary drainage procedure

The general protocol for endoscopic retrograde cholangiography (ERC) for PBD in our center is as follow: before the procedure, cross-sectional abdominal imaging is reviewed, and the location of the biliary stricture is identified. ERC is performed using a duodenoscope (TJF 160VR and TJF Q180V: Olympus Optical Co.,Ltd., Tokyo, Japan). Biliary cannulation is achieved using a sphincterotome or cannula catheter and guidewires. Biliary sphincterotomy, biliary brushing cytology, and intraductal biopsy are performed before biliary stent deployment under fluoroscopy guidance. The biliary stent(s) is placed above the stricture, and the position is confirmed by fluoroscopy. Either the 7-10 Fr straight plastic (Boston scientific, COOK and Olympus) or 10-mm covered self-expandable metal stent (Teawong [Korea], Hanaro [Korea] and Boston Scientific [USA]) is selected at the discretion of the endoscopist.

The procedure was performed under conscious sedation by five experienced endoscopists (TP, NN, JS, TY and BO). Antibiotic prophylaxis was given to all patients.

All patients were followed up as out-patient setting at 2-week after endoscopic PBD for monitoring the clinical condition, serum bilirubin level and other procedure-related complications before pancreaticoduodenectomy, this additional follow up after PBD may affect the timing for schedule the operation.

### Surgical resection procedure

The eligibility for surgery was evaluated by experienced HPB surgeons and radiologists. All patients received preoperative antibiotics for prophylaxis. The choice of curative resection, classic Whipple’s operation or PPPD, was as the surgeon’s discretion.

- PPPD was performed for tumors located around the ampulla with no evidence of invasion of the duodenum or stomach and includes the removal of all lymph nodes on the right side of the portal vein and mesenteric artery (14).

- The classic Whipple’s operation was performed if the tumor had metastasized to the proximal duodenum or pylorus and includes the resection of the distal stomach (14).

Patients were transferred to a critical care or intermediate postoperative care unit after the operation. Routine postoperative biochemical blood tests were performed. Oral intake was generally initiated after the gastric content output was ensured to be less than 500 mL/day and presence of bowel movements.

### Data collection

Data pertaining to demographic and clinical characteristics such as age, sex, tumor location, imaging results, laboratory investigations, Eastern Cooperative Oncology Group (ECOG) status, details of the endoscopic procedure (stent type, duration of the endoscopic procedure, technical details, and complications), details of the operative procedure (procedure type, blood loss, complications bleeding, bile leakage and internal organ injury), incidence of postoperative complications (intraoperative bleeding, surgical site infection, intra-abdominal collection, bile leakage, pancreatic leakage and anastomosis leakage), and date and cause of death were collected and recorded. Intraoperative bleeding was categorized according to the extent of blood loss: < 500 mL was considered normal operative bleeding, 500-1000 mL was considered mild, >/= 1000 mL was moderate and >/= 1000 mL with need for early resuscitation was considered severe.

### Statistical analysis

Patients were categorized into the PBD and DS groups. Continuous variables were compared between the two groups using Wilcoxon’s test for non-normally distributed data and student’s *t*-test for normally distributed data, whereas categorical data were compared using chi-square test or Fisher’s exact test. A p-value < 0.05 was considered statistically significant. Survival probability data were demonstrated using Kaplan-Meier survival curve and log-rank test was used for the comparison. The potential factors associated with 1-year mortality were analyzed by univariable and multivariable methods using logistic regression analyses and expressed as odds ratios (ORs) with 95% confidence intervals (CIs). All statistical analyses were performed using the R program version 4.1.0 (R foundation for statistical computing, Vienna, Austria).

## Results

During the study period, 181 patients diagnosed periampullary cancer underwent Whipple’s operation or PPPD. Of those, a total of 104 patients (58 in the PBD group and 46 in the DS group) fulfilled our eligibility criteria.

**Table 1** summarizes baseline characteristics of the patients. Age, body mass index (BMI), sex, ECOG status, presence of comorbid diseases, initial laboratory data and definite pathological diagnoses were not significantly different between the two groups. Most patients in both groups were over 60 years old but exhibited good performance status of ECOG class 1-2. The results of baseline laboratory data at diagnosis were not significantly different except for the higher median total bilirubin level, lower platelet counts, and longer prothrombin time were observed in the patients in the DS group compared with those in the PBD group. Pancreatic adenocarcinoma, ampullary adenocarcinoma, and cholangiocarcinoma were accounted for >80% of the entire cohort. All patients underwent curative resection with either Whipple’s operation or PPPD. As expected, the PBD group had a significantly longer waiting time for surgery than the DS group by approximately 30 days (p < 0.001), and the mean preoperative serum bilirubin level was respectively lower (1.8 vs 16.8 mg/dL, p = 0.001). None of the patients in the study received neoadjuvant chemotherapy, but 22.1% of them (24.1% in the PBD group, and 19.6% in the DS group) received adjuvant chemotherapy after surgical resection are shown in **Table 1**.

**Table 1:** Baseline characteristics of the patients in the study.

Variables	Preoperative biliary drainage (n = 58)	Direct surgery (n = 46)	p-value
Sex (male) #	34 (58.6)	19 (41.3)	0.119
Age (years) *	62.1± 11	65.8 ± 8.3	0.066
Body mass index (kg/m2)+	20.8 (18.9,23.7)	21.5 (19.3, 23.7)	0.101
ECOG status #			0.249
Class 1	46 (79.3)	31 (67.4)	
Class 2	12 (20.7)	15 (32.6)	
Comorbid disease #			
Cardiovascular disease	3(5.2)	1(2.2)	0.653
Chronic lung disease	2(3.4)	3(6.5)	0.653
Chronic liver disease	2(3.4)	1(2.2)	1
Neurological disease	1(1.7)	0	1
Hypertension	13(22.4)	15(32.6_	0.346
Diabetic mellitus	17(29.3)	6(13)	0.081
Hyperlipidaemia	9(15.5)	13(28.3)	0.181
Chronic kidney disease	0	3(6.5)	0.083
Laboratory finding at diagnosis			
Total bilirubin (mg/dL)+	12.7 (7, 18.3)	16.2 (9.1, 22.1)	0.049
Alanine transaminase (U/L) +	96 (51.2, 165.2)	97 (47.5,210.8)	0.751
Alkaline phosphatase (IU/L) +	451 (346.8, 674.2)	372 (275, 477)	0.029
Albumin (g/dL) *	3.6 ± 0.5	3.6 ± 0.5	0.974
Creatinine (mg/dL) +	0.8 (0.6, 0.9)	0.8 (0.6, 0.9)	0.945
Platelet count (X 10^3^) *	388.8 ± 114	336.7 ± 106	0.019
Haematocrit (%) *	33.6 ± 4.8	32.7 ± 4.2	0.297
Prothrombin time +	12.6 (12, 13.9)	14.3 (12.2, 15.8)	0.012
International normalized ratio +	1.1 (1, 1.2)	1.3 (1.1,1.5)	0.019
Type of operation #			0.446
-Whipple’s operation	12 (20.7)	6 (13)	
-PPPD	46 (79.3)	40 (87)	
Waiting duration for surgery (days)+	49 (28.2, 64.2)	19 (9.2, 29)	< 0.001
Total procedure duration (min) +	480 (420, 540)	480 (380, 540)	0.815
Preoperative serum bilirubin (mg/dL) +			
-Total bilirubin (TB), mg/dL	1.8 (0.8, 3)	16.8 (9, 22.1)	< 0.001
-Direct bilirubin (DB), mg/dL	1.4 (0.4, 2.6)	14.8 (8, 21.2)	< 0.001
Pathological diagnosis #			0.575
-Pancreatic adenocarcinoma	16 (27.6)	19 (41.3)	
-Adenocarcinoma of ampulla of Vater	27 (46.6)	19 (41.3)	
-Cholangiocarcinoma	7 (12.1)	5 (10.9)	
-Adenocarcinoma of duodenum	2 (3.4)	–	
-Neuroendocrine tumor of pancreas	1 (1.7)	–	
-Mass forming chronic pancreatitis	2 (3.4)	–	
-Cystic tumor of pancreas	3 (5.2)	3 (6.5)	
Receiving adjuvant chemotherapy	14 (24.1)	9 (19.6)	0.577

*Data are expressed as mean± SD, + Data are expressed as median (Interquartile range), # Data are expressed as n (%).

Postoperative complications and outcomes between the two groups are shown in **Table 2**. Although the time in intensive care unit, the length of hospital stays, the proportion of patients who survived less than one-year, and overall immediate complications were comparable between the two groups, interestingly, the patients in the DS group experienced a significantly higher rate of severe intraoperative bleeding (2% vs 0%) and bile leakage (15.2% vs 1.7%), respectively, and the relative risks are shown in the table.

**Table 2:** Postoperative outcomes.

Variables	Preoperative biliary drainage (n = 58)	Direct surgery (n = 46)	Relative risk (95% confidence interval)
Postoperative complications #			
- Overall complications	34 (58.6)	34 (73.9)	1.26 (0.92, 1.73)
- Intra-abdominal bleeding	17 (29.3)	15 (32.6)	1.11 (0.28, 4.42)
- Severity of bleeding (mL)	41 (75.9)	36 (78.3)	–
• < 500	13 (24.1)	8 (17.3)	reference
* 500-1000	0	2 (4.3)	0.86 (0.47, 1.56)
* >/=1000	13 (22.4)	17 (37)	2.25 (1.76, 2.87)
- Intra-abdominal collection	16 (27.6)	9 (19.6)	1.65 (0.82, 3.31)
- Surgical site infection	1 (1.7)	7 (15.2)	0.71 (0.28, 1.8)
- Bile leakage	10 (17.2)	13 (28.3)	8.83 (1.26, 61.79)
- Pancreatic leakage	2 (3.4)	2 (4.3)	1.64 (0.68, 3.94)
- Small bowel injury -			
Need for re-operation #	7 (12.1)	3 (6.5)	0.54 (0.08, 3.81)
Length of intensive care unit (days) *	6.4 (4.4)	5.7 (4.1)	–
Length of hospital stay (days)+	11.5 (10,17.8)	13.5 (10, 23.2)	–
Death within one year #	12 (20.6)	16 (34.7)	1.45 (0.86, 2.45)

* Data are expressed as mean ± standard deviation, + Data are expressed as median (interquartile range), # Data are expressed as n (%).

**Figure 1:**
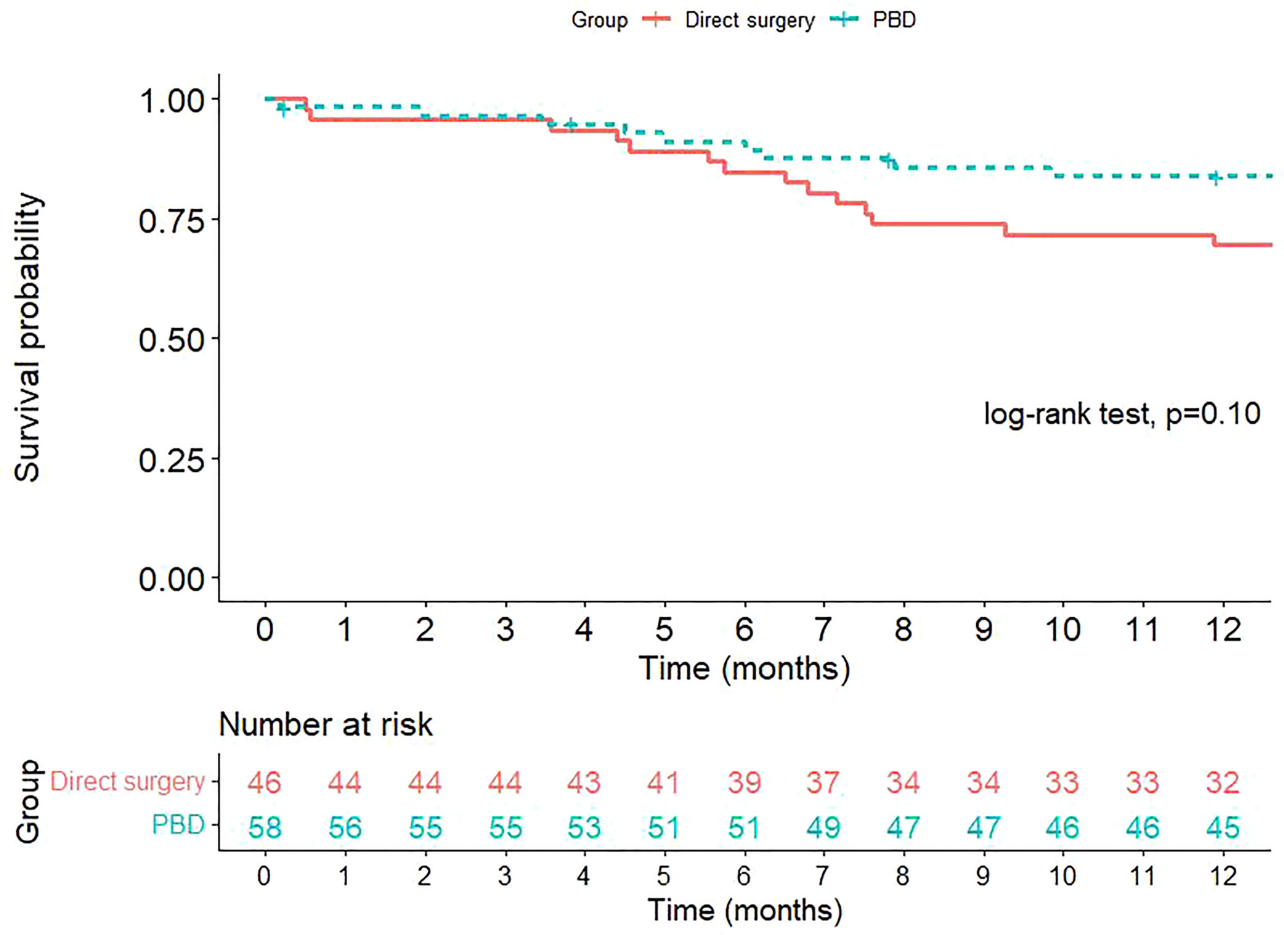
Kaplan-Meier survival curve for overall survival after pancreaticoduodenectomy.

The overall survival of the patients in both groups are presented in **Figure 1**. At one-year after surgery, 20.6% of the patients in the PBD group and 34.7% of the patients in the DS group deceased. However, the difference in overall survival between the two groups was not statistically significant.

**Figure 2:**
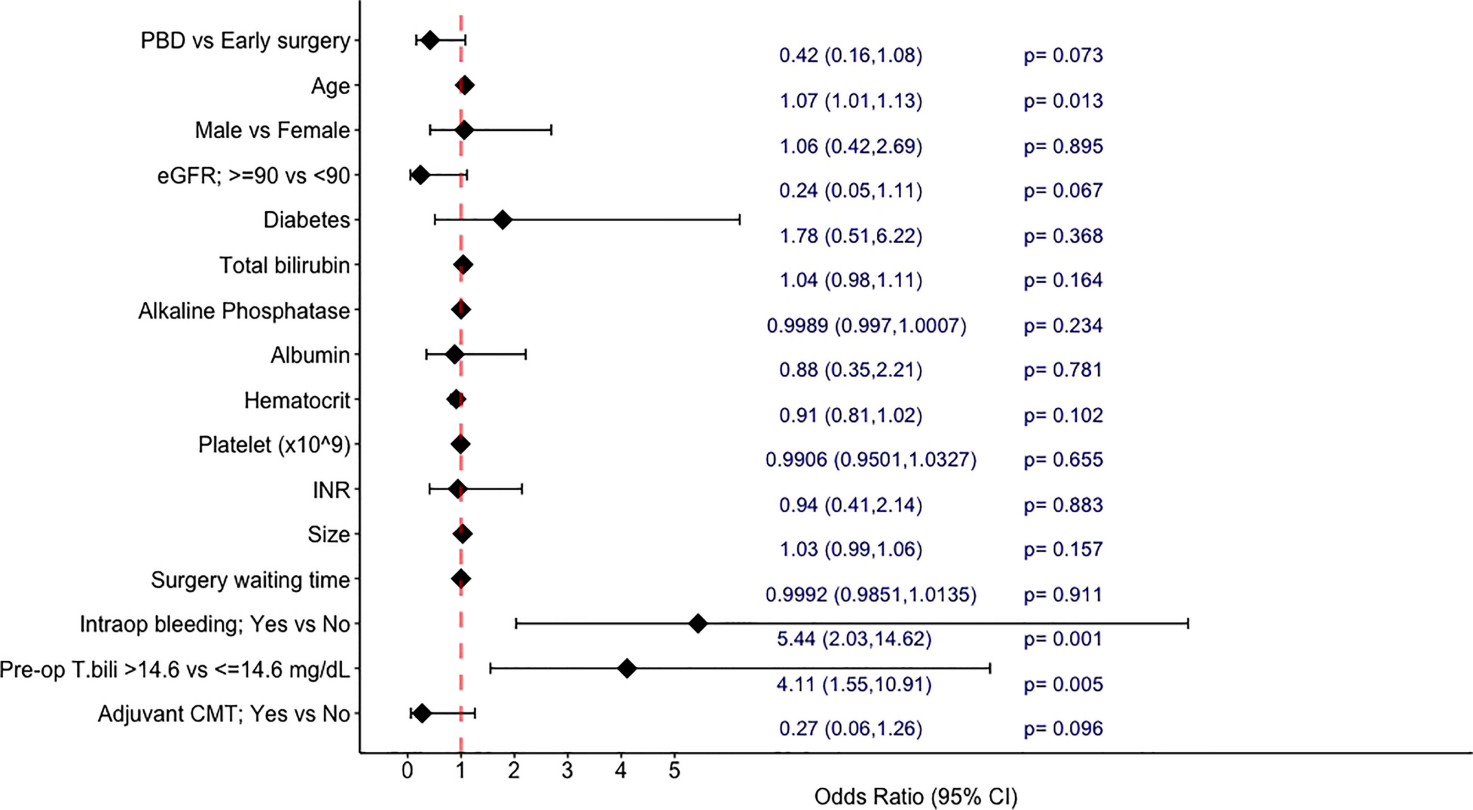
Univariable analysis for factors associated with mortality.

In the univariable analyses exploring the factors associated with 1-year mortality (**Figure 2**), PBD showed a trend towards a lower 1-year mortality rate with an odds ratio (OR) of 0.42 (95%CI: 0.16-1.08, p=0.073). Other factors associated with increased risk of 1-year mortality by univariate analyses were increasing age (OR 1.07; 95%CI 1.01-1.13, p=0.013 per year), preoperative bilirubin level of higher than 14.6 mg/dL (OR 4.11; 95%CI 1.55-10.91, p=0.005), and presence of intraoperative bleeding (OR 5.44; 95%CI 2.03-14.62, p<0.001). Having normal creatinine clearance (>/=90 ml/min/1.73m^2^) at the time of diagnosis and receiving adjuvant chemotherapy were additional factors that showed trends towards a lower risk of death within 1 year (p<0.1). Sex, diabetes, degree of jaundice at the time of diagnosis, tumor size, surgery waiting time, and other laboratory data were not associated with one-year mortality.

**Figure 3:**
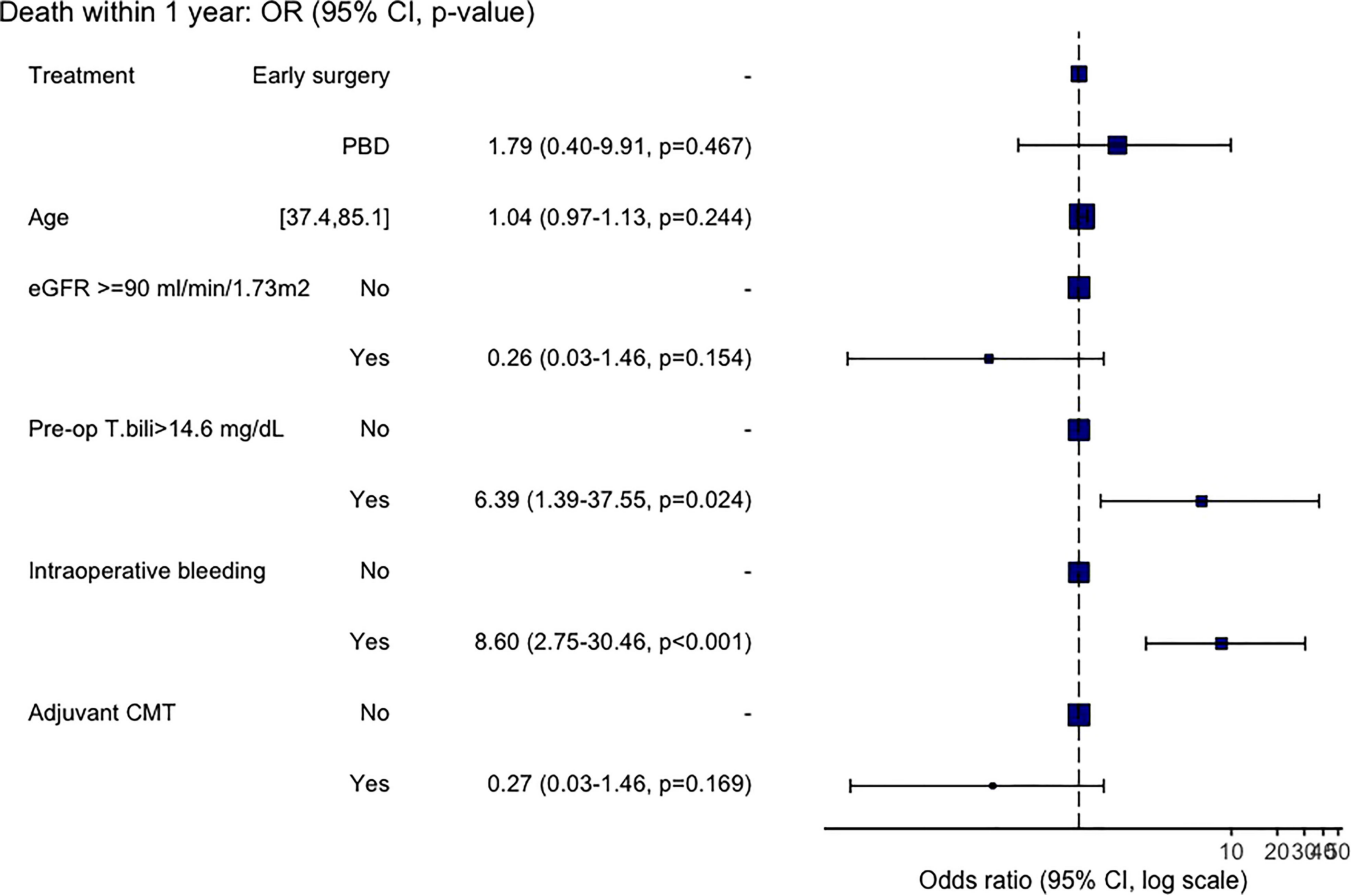
Multivariate analysis for factor associated with mortality.

All variables with p<0.1 from the univariable analyses were then entered to the multivariable analysis and the results are shown in **Figure 3**. From the multivariable analysis, only intraoperative bleeding, and preoperative bilirubin level of >14.6 mg/dL were independent predictors for death within 1-year with an adjusted OR of 8.60 (95%CI: 2.45-30.46, p<0.001) and 6.39 (95%CI: 1.39-37.55, p<0.001), respectively. While age, normal creatinine clearance, PBD, and adjuvant chemotherapy recipients were not independently associated with 1-year mortality.

## Discussion

Pancreaticoduodenectomy is a high-risk surgery, associated with high morbidity in patients with periampullary cancer, but also is an only curative treatment option (15). Adequate preoperative preparation of patients undergoing PD is crucial to minimize adverse outcomes. The frequency of complications is higher in patients with severe obstructive jaundice, malnutrition, and cholangitis; therefore, PBD is theoretically useful in such patients (16, 17). Two approaches for PBD, endoscopic and percutaneous, are generally used for biliary decompression in patients with periampullary cancer presenting with obstructive jaundice in clinical practice (1, 4). Nonetheless, whether patients with obstructive jaundice should go for direct surgery or PBD first is still debatable.

Our study demonstrates that, in resectable periampullary cancer patients, PBD did not show a survival benefit in comparison to DS. However, those who underwent PBD had a lower rate of immediate postoperative complications namely bile leakage, and degree of intraoperative bleeding. And having intraoperative bleeding, and preoperative bilirubin over 14.6 mg/dL were independent predictors associated with an increased risk of death within one year.

The baseline characteristics of the PBD and DS group were generally similar in this study. The median total bilirubin level at the diagnosis and INR were slightly higher, and the platelet count was lower in the DS group compared to the PBD group. Nonetheless, these 3 variables were not significantly associated with 1-year mortality. Of note, serum bilirubin level at the diagnosis in our study (mean 14.3 ± 7.8 mg/dL) was higher than that reported in other studies (1, 18), which may represent the late presentation or the longer time to diagnosis of patients in this study. Moreover, the waiting time for surgery in the present study (median 30 days; IQR 15-53.5 days) was quite longer than that reported in previous studies (1-2 weeks) even none of the patients received neoadjuvant chemotherapy (1, 19), which reflects the situation in developing countries where the healthcare system is usually overwhelmed.

The mean preoperative bilirubin level sharply declined from 12.7 to 1.8 mg/dL within 2 weeks after endoscopic biliary drainage in the PBD group, indicates the adequacy of biliary decompression, according to the European Society of Gastrointestinal Endoscopy (ESGE) guidelines (20). Adequate PBD in patients with periampullary cancer has been reported benefit in reducing the occurrence of major morbidities (38.9% vs 61.1%) postoperatively compared to inadequate drainage (18). This might support our results PBD advantages in terms of the lower rates of intraoperative bleeding and bile leakage compared with the DS group. And it is also in concordant with the result from a previous meta-analysis that patients with resectable malignant distal biliary obstruction who had undergone internal PBD had significantly lower incidence of major postoperative complications than those who had undergone DS (9), and the recent retrospective study in patients with severe jaundice that the fewer cases of post-pancreatectomy hemorrhage was observed in the PBD group (21). In addition, endoscopic approach is less invasive than percutaneous approach for PBD (22). The benefit of significantly lower intraoperative bleeding and postoperative bile leakage in the PBD group than in the DS group may be explained by the regained coagulation function, as effective biliary drainage leads to an improvement of vitamin K absorption; and biliary decompression could downsize the dilated bile duct and might result in a lower chance of bile duct injury during surgical procedures.

The choice of biliary drainage stent type was controversy, previous reports showed self-expanding metal stents (SEMs) were associated with lower risks of post procedural cholangitis (4.1% VS 9.7%, p=0.043) and fewer postoperative pancreatic fistula (9.8% vs 18.5%, p = 0.004) than plastic stents (23). However, a recent meta-analysis showed comparable postoperative outcomes between metal and plastic stents (24). In this cohort, plastic stents were used in most patients (>95%) according to the national reimbursement policy, making the further analysis regarding stent subtype was not possible in our study.

On the contrary, studies have reported the negative impact of PBD on postoperative outcomes in terms of increased incidence of infectious complications and bleeding (at a relative risk [RR] of 1.66; 95% CI, 1.28-2.16; p = 0.0002) (1, 6, 25). Garcea et al, showed that PBD was associated with a significantly increased probability of wound infection (OR, 1.827; p < 0.005) (8). Nonetheless, these negative outcomes were not observed in our cohort. The routine prophylactic antibiotic prior to the operation in our study might play a role in this finding.

In the present study, the one-year overall mortality was 20.6% in the PBD group and 34.7% in the DS group, while the probability of postoperative death within one-year was slightly higher in the DS group than in the PBD group, the difference was not statistically significant (p = 0.107). The lack of benefit of PBD in lowering mortality has also been shown in previous reports (1, 6, 9).

We also evaluated the factors associated with 1-year mortality after curative surgical resection. As mentioned earlier, PBD itself was not associated with a better 1-year survival. After adjustment with many potential factors, we found that only pre-operative serum bilirubin level of >14.6 mg/dL, not the bilirubin level at the time of diagnosis, was significantly associated with a higher risk of death within one-year with an adjusted OR of 6.39 (95%CI: 1.39-37.55, p<0.001). And the other independent factor showed an increased risk of 1-year mortality was having intraoperative bleeding (an adjusted OR of 8.60 (95%CI: 2.45-30.46, p<0.001)), whereas the development of pancreatic fistula and bile leak were not significantly associated with the poorer survival outcome. These are interesting findings that may highlight the potential role of PBD in a subgroup of patients. As prior studies that showed no beneficial effect (or negative impact) of PBD on postoperative outcomes were mainly studied in patients with a lower level of bilirubin; for instance, the RCT by van der Gaag, et al. (1) included only patients with baseline bilirubin level of lower than 250 μmol per liter (<14.6 mg/dL), and the large retrospective study by de Pastena, et al. most of the patients had bilirubin level of less than 10.2 mg/dL (26).. Moreover, the recent article studied for the overall mortality also demonstrated that the higher level of total bilirubin before surgery (over 150 μmol per liter – about 8.77 mg/dL) was associated with a lower risk of overall survival (27).

Adjuvant chemotherapy is another interesting factor, receiving postoperative chemotherapy was associated with a better outcome in the univariate analysis, but not in the multivariate analysis in our study, yet the towards a better 1-year survival was still observed (adjusted OR 0.26, p=0.169). The data regarding the benefit of adjuvant chemotherapy in these patients are still controversial, a prospective study (ESPAC-3) showed the survival advantage of the adjuvant chemotherapy (a combination of fluorouracil and folinic acid or gemcitabine) with a hazard ratio of 0.75 (95% CI 0.57-0.98, p = 0.03) (28). while in a retrospective study of patients with resectable pancreatic adenocarcinoma, the use of adjuvant chemotherapy (FU-based or gemcitabine-based) was not associated with improved long-term survival (p=0.69) (29). A study with a larger sample size may demonstrate the benefit of adjuvant chemotherapy more clearly.

This study represents a real-world situation of resectable periampullary cancer patients in developing countries, in which the presentation of the patients is usually late as severe jaundice was commonly observed, and the waiting time before surgery was quite long. We found no deleterious effect of PBD compared to DS, and some beneficial postoperative outcomes were also observed. The limitations of our study are noted. As it is retrospective in nature, some differences in baseline characteristics of the patients in the PBD and the DS groups existed, however, those differences in baseline laboratory data were not associated with the outcomes in our study. In addition, a significant proportion of the patients were referred back to their local hospital after surgical resection and being followed-up for over a year, this makes it is difficult to evaluate the disease-free survival and long-term (e.g., 3-year, or 5-year) survival of the patients in our study.

## Conclusions

PBD in resectable malignant distal biliary obstruction showed no benefit in terms of 1-year survival over DS approach. But it demonstrated the benefit of lower risks of intraoperative bleeding, and bile leakage. Additionally, the level of pre-operative bilirubin level of over 14.6 mg/dL and having intraoperative bleeding were associated with a lower 1-year survival in such patients. Overall, PBD may be not necessary for all resectable periampullary cancer patients, but there might be a role in those with severely jaundice (>14.6 mg/dL), as it helps lower risk of intraoperative bleeding, and might lead to a better survival outcome.

## Data availability statement

The original contributions presented in the study are included in the article/supplementary material. Further inquiries can be directed to the corresponding author.

## Author contributions

TPat has made substantial contribution to the conception and design of the study, data collection as well as manuscript writing. TC and PS have made contributions to the design of the study, data analysis and manuscript writing. NN, BO, TY, TP and SA performed and completely reported endoscopic and surgical data. PS, NC and SA have made contributions to manuscript writing and English language approval. TPat is the first author. PS is the corresponding author, and responsible for ensuring that all listed authors have approved the manuscript before submission. All authors contributed to the article and approved the submitted version.

## Funding

The study was supported by Faculty of Medicine, Prince of Songkla University.

## Acknowledgments

The authors thank Faculty of Medicine, Prince of Songkla University, for providing resource and aiding data collection in the study. We also thank all patients and hope this work will be beneficial for the clinical practice in the future.

## Conflict of interest

The authors declare that the research was conducted in the absence of any commercial or financial relationships that could be construed as a potential conflict of interest.

## Publisher’s note

All claims expressed in this article are solely those of the authors and do not necessarily represent those of their affiliated organizations, or those of the publisher, the editors and the reviewers. Any product that may be evaluated in this article, or claim that may be made by its manufacturer, is not guaranteed or endorsed by the publisher.

## Abbreviations

CI, Confidence interval; CT, Computed tomography; ECOG, Eastern Cooperative oncology Group; ERC, Endoscopic retrograde cholangiography; ESGE, European Society of Gastrointestinal Endoscopy; HPB, Hepato-pancreato-biliary; MRI, Magnetic resonance imaging; OR, Odd ratios; PBD, Preoperative biliary drainage; PD, Pancreaticoduodenectomy; PPPD, Pyloric-preserving pancreaticoduodenectomy; 5FU, fluorouracil; LV, leucovorin.
